# Estimating smoking-attributable lung cancer mortality in Chinese adults from 2000 to 2020: a comparison of three methods

**DOI:** 10.1186/s12885-023-11661-0

**Published:** 2024-01-09

**Authors:** Feiling Ai, Jian Zhao, Wenyi Yang, Xia Wan

**Affiliations:** Institute of Basic Medical Sciences, Chinese Academy of Medical Sciences/School of Basic Medicine, Peking Union Medical College, Beijing, 100005 China

**Keywords:** Population attributable fraction, Smoking impact ratio, Lung cancer, Dose–response relationship

## Abstract

**Background:**

Smoking is a significant public health concern in China and a leading cause of lung cancer deaths among adults. This study aims to employ three methods to estimate smoking-attributable lung cancer mortality among Chinese adults from 2000 to 2020.

**Methods:**

Population attributable fractions (PAFs) of lung cancer deaths caused by smoking were estimated using lagged smoking prevalence, Peto-Lopez, and dose–response relationship methods, separately. Smoking exposure was obtained from national tobacco surveys in China, and relative risks (RR) were derived from a meta-analysis of state-of-the-art studies among the Chinese population. Finally, we estimated the sex- and age-stratified smoking-attributable lung cancer deaths in Chinese population in 2000, 2005, 2010, 2015, and 2020.

**Results:**

The PAFs estimated using 5- and 10-year lagged smoking prevalence method (45–47%) and Peto-Lopez method (46–47%) were similar, while PAFs calculated using the dose–response method were highest (47–58%). The PAFs were consistently higher in males than in females. Age-specific PAFs estimated by lagged smoking prevalence method (54–60%) and the Peto-Lopez method (57–61%) in males were similar and relatively stable, with slight decreases in older populations, while the dose–response relationship-based PAFs increased with age and fluctuated by year. By using the above methods, smoking-attributable lung cancer deaths were estimated to be 134,100, 134,600, 136,600, and 155,300 in 2000 increasing to 310,300, 301,100, 306,000, and 314,700 in 2020, respectively.

**Conclusion:**

The estimation from dose–response methods could better reflect the smoking effect, however, high-quality data and accurate estimation of parameters are necessary.

- What is already known on this topic – the dichotomous relative risk (RR) along with the lagged smoking prevalence or smoking impact ratio (SIR) are widely used in estimating smoking-attributable disease burden and recently the Global Burden of Disease (GBD) study group employed a dose–response RR based method to estimate smoking-attributable deaths.
- What this study adds – this is the first study using three major methods to estimate smoking-attributable lung cancer mortality in China at the same time and comparing the methods differences, and the study revealed that smoking-attributable lung cancer mortality estimates in China varied across three methods, with the highest estimates derived from the dose–response relationship method.
- How this study might affect research, practice or policy – the findings of this study indicate that the dose–response method in country level could offer more reasonable estimation of disease burden and provide more useful evidence for tobacco control.

## Introduction

Tobacco use is a leading driver of a wide range of diseases including cancer, respiratory and cardiovascular diseases and premature death [1]. Annually, around 8 million deaths and 1.4 trillion USD in economic losses are attributable to tobacco use worldwide [2]. Tobacco use is also the largest preventable cause of death, and effective tobacco control could significantly reduce the burden of chronic diseases and global deaths by 71% [3]. China is a major producer and consumer of tobacco with more than 300 million smokers, and the prevalence among male adults is 50.5% [4]. Despite continuous efforts put into tobacco control, smoking remains a significant public health threat in China. Accurate estimation of disease burden caused by tobacco use is crucial to resource allocation, policy making and dissemination.

Currently, there are three major methods used to estimate the population attributable fraction (PAF) [5] of smoking-attributable deaths – lagged smoking prevalence method [6, 7], Peto-Lopez method [8] and the dose–response relationship method [9]. Based on these methods, Global Burden of Disease (GBD) studies estimated that in China, there were 0.59, 1.21, 1.59, 1.77, 2.20, and 2.42 million all-cause deaths could be attributable to smoking in 2000 [10], 2010 [11], 2013 [12], 2015 [13], 2017 [14], and 2019 [9], showing a continuous increase especially in the most recent estimates.

The lagged smoking prevalence method and Peto-Lopez method were widely used in many years and both methods calculate PAF using dichotomous relative risk (RR). Since 2017, GBD study group has been using a dose–response RR-based method to estimate the smoking caused disease burden. They hold that the dose–response method could better capture differences in risk from heterogenous smoking patterns as it utilized more dimensional parameters of smoking, including smoking amounts, years of smoking or quitting, instead of "yes or no" [15]. Up to now, lack of studies to compare these methods and discuss their application scenarios. Moreover, there is a sharp increase of the estimated deaths in China, which has also aroused many doubts among Chinese government and experts in tobacco control area.

Therefore, it is necessary to estimate disease burden caused by smoking in China using three methods respectively and further validate whether the estimations in China are reasonable.

In this study, we calculated the smoking-attributable PAFs and lung cancer deaths in Chinese adults in 2000, 2005, 2010, 2015, and 2020 taking into account that lung cancer is the most extensively studied disease [16] among all smoking-related causes with comparatively sufficient evidence. We used same data source and then compared estimates derived from the 5- and 10-year lagged smoking prevalence method, Peto-Lopez method and dose–response relationship method.

## Methods

### Data sources

The age- and sex-specific smoking prevalence was obtained from the China national surveys—Chinese Adult Tobacco Surveys in 1984 [17], 1996 [18], 2010 [19], 2015 [20], and 2018 [21], and Chinese Chronic Disease and Risk Factors Surveillance (CCDRFS) in 2002 [22] and 2007 [23]. Age- and sex-stratified pack-years (PYs) were extracted from 1996, 2002, 2010 and 2018 surveys, while the quit-years (QYs) were obtained from the 2010 and 2018 surveys due to data availability. The dichotomous RRs and the dose–response relationship RRs function were obtained from a meta database of smoking and attributable diseases in the Chinese population as of June 30th 2021 [24]. Age- and sex-specific lung cancer mortality were obtained from Chinese mortality surveillance database of 2000, 2005, 2010, 2015, and 2020. Age- and sex-specific population data were obtained from the fifth (2000), sixth (2010) and seventh (2020) national censuses [25] and statistical yearbooks of 2006 and 2016 [26].

### Estimating smoking exposure

Smoking exposure includes ever, current and former smoking prevalence. Ever smoking prevalence means the percentage of adults who have ever smoked any tobacco in their lifetime in the total population. Current smoking prevalence was defined as the percentage of adults who smoked tobacco at the time of interview in the adult population. Former smoking prevalence refers to the percentage of adults who smoked in the past but were no longer smoking at the time of investigation.

Smoking definition was stricter in 1996 and 2002 surveys than that in other surveys, by setting the threshold of 100 cigarettes for life, which may underestimate the smoking prevalence by 1–3% [27]. Therefore, ever and current smoking prevalence in 1996 and 2002 increased by 1% with the conservative assumption. The sex- and 5-year-age-specific smoking prevalence started at 15-year-old. Smoking prevalence and quitting proportions in 1990, 1995, 2000, and 2005 were obtained by fitting with a spline function. Smoking prevalence in 2020 was substituted by that of 2018. Due to the lagged effects of smoking, both 5- and 10-year lagged smoking prevalence were used in the formula [11].

The smoking impact ratio (SIR) which acts as a useful indirect measure of accumulative smoking exposure were estimated according to the following steps. First, the non-smoker lung cancer mortality rate (N) of the study population was calculated:

$$\mathrm{N}=\frac{C}{\mathrm{P}\times \mathrm{RR}+(1-\mathrm{P})}$$

Then, SIR was calculated using the following formula adjusted by Ezzati [28] et al.:

$$\mathrm{SIR }= \frac{\mathrm{C }-\mathrm{N}}{{\mathrm{S}}^{*} - {\mathrm{N}}^{*}} \times \frac{{\mathrm{N}}^{*}}{\mathrm{N}}$$

*RR is the dichotomous RR (female: 3.18, male: 3.26)* [29]*;*

*P is the smoking prevalence;*

*C is the lung cancer mortality rate;*

*N is the lung cancer mortality rate among nonsmokers in the study population;*

${S}^{*}$* and *${N}^{*}$* are the lung cancer mortality rates among smokers and nonsmokers in the reference population, which is the US CPS-II cohort* [30]*.*

Missing age groups were replaced using adjacent values or mean values. The maximal SIR was limited to 1 to avoid overestimation [31].

### Smoking exposure distribution

In order to identify the different risks of disease within the groups based on the smoking intensity and length of time since cessation, we estimated the distribution of cumulative PYs across smokers’ lifetime among current smokers and years since cessation among former smokers (QYs).

For current smokers, the 5-year and sex-specific PYs were extracted among adults aged 30 years and above from 1996, 2002, 2010 and 2018 surveys by multiplying average daily cigarette and years of smoking. For former smokers, the age- and sex-stratified QYs were only obtained from the combination of the 2010 and 2018 surveys. Then individual-based sampling data were weighted to represent the national level and frequency table and probability density functions of PYs and QYs have fitted accordingly. In the fitting process, normal, lognormal, beta, weibull, logistic, exponential, and power functions were fitted in turn, and the best-fitting probability density function was chosen based on the decreasing chi-square. The lag period of PYs was set as 5 years, which means that the exposure distributions of 1996, 2002, 2010, and 2018 were used to substitute the years of 2000, 2005 and 2010, 2015, and 2020, respectively.

### Relative risk of lung cancer caused by smoking

The dichotomous RRs and 95% CI for lung cancer in male and female smokers in China were 3.26 (2.79–3.82) and 3.18 (2.78–3.63), respectively [29].

The RR per unit of SIR was calculated as:

$${\mathrm{RR}}_{\mathrm{SIR}} = \frac{\mathrm{C }-\left(1 -\mathrm{SIR}\right) \times \mathrm{N}}{\mathrm{SIR }\times \mathrm{N}}$$

*C is the lung cancer mortality rate in the study population;*

*N is the lung cancer mortality rate among nonsmokers in the study population;*

*SIR is the smoking impact ratio.*

The dose–response RRs were estimated based on the integrated exposure–response (IER) model with the expression: $y=1+89.95\times (1-{e}^{-0.001\times x})$ [24].

### The estimation of PAF

The PAFs of lung cancer deaths caused by smoking in Chinese adults aged 30 years and above in 2000, 2005, 2010, 2015 and 2020 were estimated by employing lagged smoking prevalence method, the Peto-Lopez method and the dose–response relationship method, respectively.

1. Lagged smoking prevalence method:

$$PAF=\frac{P\times (RR-1)}{P\times (RR-1)+1}$$

*P is the lagged smoking prevalence and RR is the dichotomous RR*.

(2) Peto-Lopez method:

$$PAF=\frac{SIR\times ({RR}_{SIR}-1)}{SIR\times ({RR}_{SIR}-1)+1}$$

*SIR is the smoking impact ratio and* ${RR}_{SIR}$ *is the RR per unit of SIR back-calculated*.

(3) Dose–response relationship method [9]:

$$\mathrm{PAF }= \frac{\mathrm{P}\left(\mathrm{n}\right) +\mathrm{P }\left(\mathrm{c}\right) \int \mathrm{exp}\left(\mathrm{x}\right)\mathrm{RR}\left(\mathrm{x}\right)+\mathrm{P}\left(\mathrm{f}\right)\int \mathrm{exp}\left(\mathrm{y}\right)\mathrm{RR}\left(\mathrm{y}\right) -1 }{\mathrm{P}\left(\mathrm{n}\right) +\mathrm{P}\left(\mathrm{c}\right) \int \mathrm{exp}\left(\mathrm{x}\right)\mathrm{RR}\left(\mathrm{x}\right) +\mathrm{P}\left(\mathrm{f}\right) \int \mathrm{exp}\left(\mathrm{y}\right)\mathrm{RR}\left(\mathrm{y}\right)}$$

*P(n) is the rate of non-smoking, RR = 1;*

*P(c) is the rate of current smoking, exp(x) is the distribution of PYs, and RR(x) is the dose–response RR corresponding to current smokers in different PYs;*

*P(f) is the proportion of quitting smoking, exp(y) is the distribution of QYs, and RR(y) is the dose–response RR corresponding to quitters in different QYs.*

### Estimating attributable deaths

We used the crude death rates (CDRs) to calculate the lung cancer deaths in the Chinese population. Then, the age-, sex- and year-stratified, smoking-attributable lung cancer deaths were calculated based on PAFs and lung cancer deaths [28] using the formula below:

$${\mathrm{A}}_{\mathrm{y},\mathrm{a},\mathrm{s}}={\mathrm{PAF}}_{\mathrm{y},\mathrm{a},\mathrm{s}}\times {\mathrm{D}}_{\mathrm{y},\mathrm{a},\mathrm{s}}$$

*A is the attributable lung cancer deaths; D is the number of lung cancer deaths;*

*y, a, and s denote year, age group, and sex, respectively.*

Lastly, smoking-attributable lung cancer deaths among all age groups and both sexes were combined to calculate the total attributable deaths. And the 5-year overall PAFs were calculated by dividing total population in 2000, 2005, 2010, 2015, and 2020.

SAS 9.4 was used for statistical analysis; Origin Pro 9.1 was used to fit the probability density distribution function.

## Results

### Smoking prevalence

**Fig. 1:**
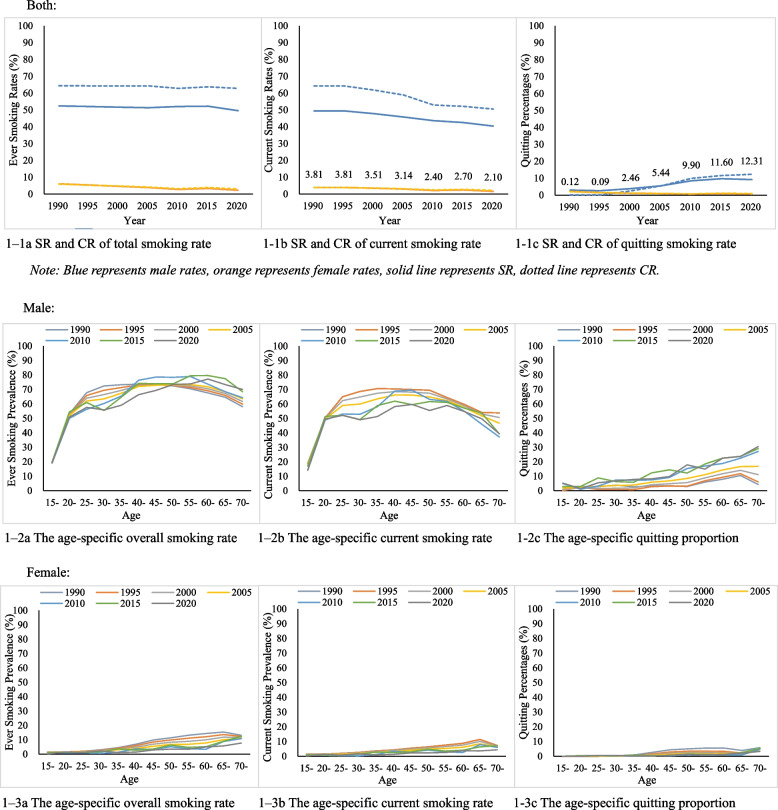
The age- and sex- specific crude rate (CR) and standardized rate (SR) of smoking and quitting in the Chinese population, 1990–2020. Both: 1–1a SR and CR of total smoking rate. 1-1b SR and CR of current smoking rate. 1-1c SR and CR of quitting smoking rate. Male: 1–2a The age-specific overall smoking rate. 1–2b The age-specific current smoking rate. 1-2c The age-specific quitting proportion. Female: 1–3a The age-specific overall smoking rate. 1–3b The age-specific current smoking rate. 1-3c The age-specific quitting proportion

Figure 1 shows the age- and sex- specific crude rate and standardized rate of smoking and quitting in the Chinese population from 1990 to 2020. Among males, ever smoking prevalence has remained stable over the past 30 years, fluctuating between 62.81% (2020) ~ 64.37% (1990), with age-standardized prevalence stabilizing at around 50%; and the current smoking prevalence decreased from 64.25% in 1990 to 50.50% in 2020, and the age-standardized prevalence also showed a decreasing trend; the smoking cessation proportion was less than 3% before 2000 and showed a gradual increase after 2000, reaching 12.31% in 2020. In contrast, among females, the ever and current smoking prevalence was much lower, with a decreasing trend from the view of standardized smoking prevalence The crude ever and current smoking prevalence of females was 5.86% and 3.81% in 1990 and 2.96% and 2.10% in 2020, respectively (figure 1-1).

**Fig. 2:**
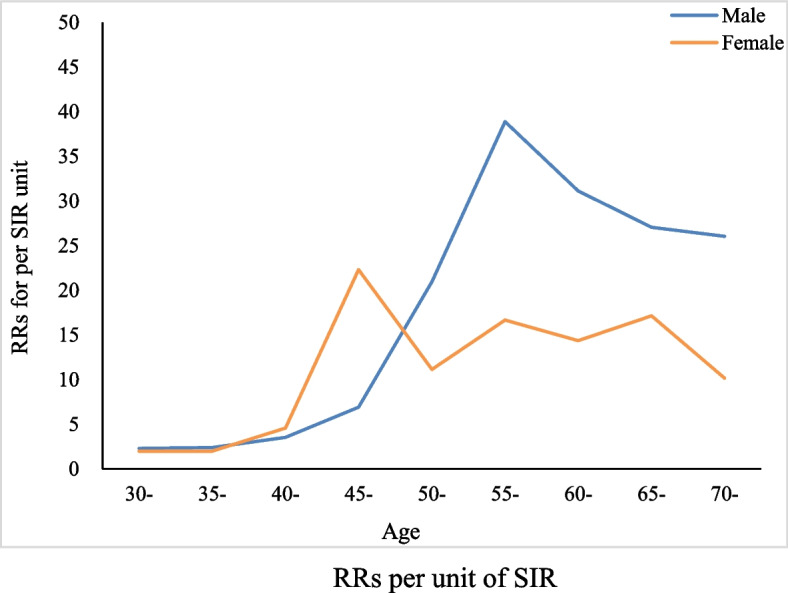
RRs per unit of SIR

In terms of age, ever smoking prevalence among males peaked at 40 years old (around 73%) before 2000 and the peak age continued to move backward, with age 65 peaking value at about 80% in 2015. Ever smoking prevalence among females was much lower before 30 years old and then increased slightly, reaching the maximum of 10–12% at 65 years old. The current smoking prevalence represented similar trends as ever smoking. Quitting proportion among the general population increased instantly by age (Fig. 1–2).

### SIRs

The male SIRs did not vary much by year but fluctuated significantly among different age groups. The SIRs were adjusted to 1 for the 30–39 age group, and fluctuated between 56.65% and 65.23% among the 40–44 age group, then decreased to 25.39–28.77% for 45–49 age group, and after 55 years old, SIR stabilized between 4.00% and 6.50%.

The female SIRs decreased by year, except for 2010 with the lowest values. Before 45 years old, the SIRs were relatively high, and the lowest SIRs were found between the 45–49 age group (Table 1).

**Table 1:** Age- and sex-specific SIR values (%) based on CPS-II during 2000–2020

Sex	Age, years	Year				
		2000	2005	2010	2015	2020
Male						
	30–34	100	100	100	100	100
	35–39	100	100	100	100	100
	40–44	62.02	61.57	65.23	63.38	56.65
	45–49	26.73	26.66	28.77	27.04	25.39
	50–54	7.89	7.92	8.48	7.99	7.94
	55–59	4.13	4.18	4.50	4.53	4.21
	60–64	5.05	5.15	5.28	5.71	5.53
	65–69	5.57	5.67	5.67	6.42	6.08
	≥ 70	5.32	5.48	5.55	5.90	6.03
Female						
	30–34	5.21	4.60	1.26	3.20	4.92
	35–39	7.63	6.47	2.97	8.07	2.39
	40–44	3.00	2.40	2.22	1.69	0.87
	45–49	0.71	0.56	0.34	0.37	0.30
	50–54	1.78	1.45	1.10	1.35	0.75
	55–59	1.25	0.96	0.52	0.66	0.47
	60–64	1.63	1.27	0.56	0.85	0.81
	65–69	1.60	1.36	1.19	1.21	0.78
	≥ 70	2.78	2.61	2.56	2.95	1.83

### RR of per unit RR

The RR per unit of SIR ranged from 2.30 to 38.89 for males and 2.00 to 22.32 for females across age groups. The RR per unit of SIR showed an increase at first and then decreased to a stable level as age increased. The maximum values of males and females were shown at 55 (38.89) and 45 (22.32) years old, respectively (Fig. 2).

### Distribution of PYs and QYs

The distribution of PYs was similar in different years. Most current smokers smoked 0–20 PYs, with 65.38%, 64.34%, 57.26% and 50.02% for males and 74.64%, 79.51%, 70.16% and 62.76% for females in 1996, 2002, 2010 and 2018, respectively. Among them, 0–10 PYs (about 25–35%) and 10–20 PYs (about 25–30%) were predominantly for males and 0–10 PYs (about 50%) for females. In terms of the year, both female and male current smokers showed a decreasing proportion of low PYs (0–20 PY) and an increasing proportion of high PYs (≥ 20 PY). For different age groups, PYs increased by age, and total amounts in males were always higher than females.

**Fig. 3:**
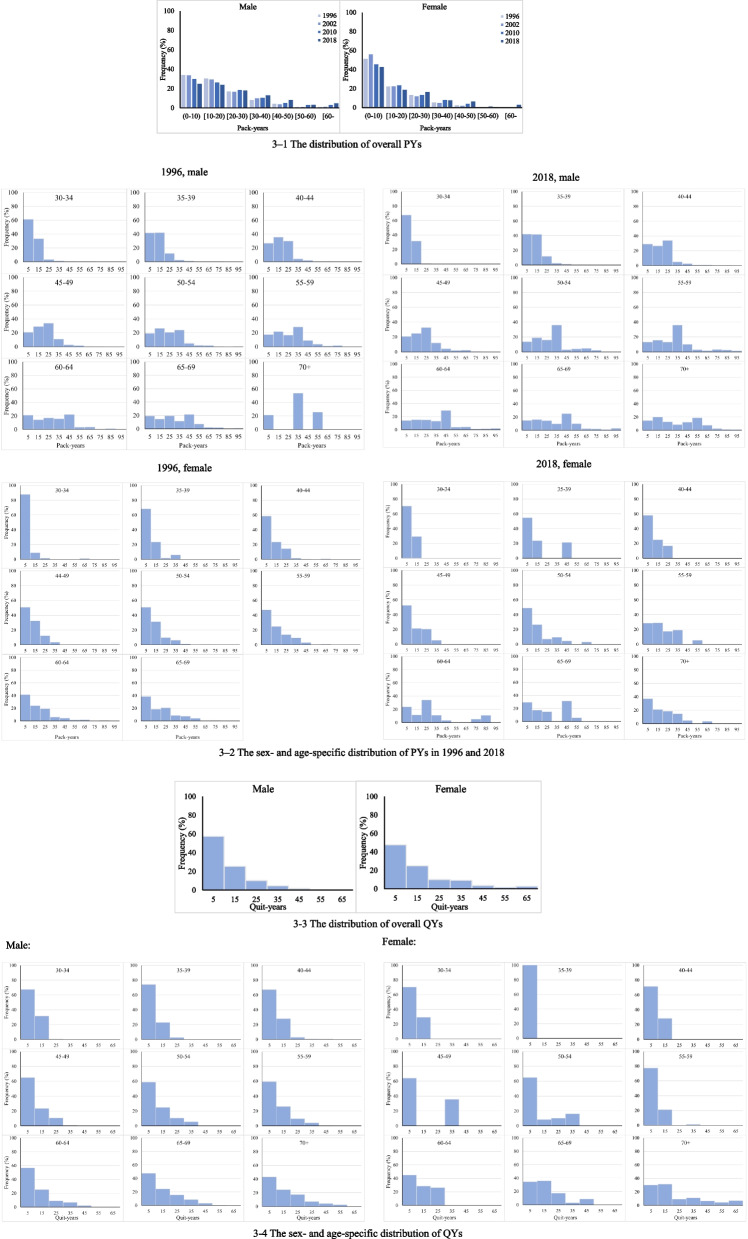
The distribution of PYs and QYs in the Chinese population. 3–1 The distribution of overall PYs. 3–2 The sex- and age-specific distribution of PYs in 1996 and 2018. 3–3 The distribution of overall QYs. 3–4 The sex- and age-specific distribution of QYs

The QYs distribution was similar in males and females, with largest proportion in 0–10 and 10–20 years. 57.42% males and 47.58% females quitted for 0–10 years. The overall QYs increased by age (Fig. 3).

The age-, sex, and year-specific probability density functions of PYs were mainly Logistic, GaussAmp, Allometric, Boltzmann, and ExpDec functions; and for QYs, they were Allometric, ExpDec, GaussAmp, and Logistic functions.

### The total PAFs

The total PAF calculated by 5-year-lagged and 10-year-lagged smoking prevalence method (45–47% and 45–57%) and the Peto-Lopez method (46–47%) were similar, however, the total PAF derived from dose–response relationship method was higher (47–58%) than the values calculated from the above two methods.

The male PAFs were constantly higher than female PAFs among the three methods. In males, the PAFs of smoking-attributable lung cancer deaths based on the lagged smoking prevalence method, the Peto-Lopez method and the dose–response relationship method were 57–59%, 59–61% and 56–72% for males, and 15–22%, 12–18% and 6–13% for females, respectively.

In terms of the year, PAFs estimated by lagged smoking prevalence method and the Peto-Lopez method did not vary a lot, whereas the dose–response relationship method fluctuated slightly between 2010 and 2020, with an increasing trend at first and then decreasing, showing the lowest value in 2015; in contrast, females had the highest value in 2015 (Table 2).

**Table 2:** PAF (%) of the smoking population estimated based on the three methods

Sex	Year	Lagged smoking prevalence method		Peto-Lopez method	Dose–response method
		5-year lagged	10-year lagged		
Male					
	2000	57.03	56.59	59.02	72.36
	2005	57.33	56.87	59.36	71.11
	2010	57.66	57.18	59.83	71.06
	2015	58.25	57.72	61.21	56.25
	2020	59.36	57.98	60.78	65.26
Female					
	2000	20.05	21.78	18.22	8.95
	2005	18.42	20.17	16.57	8.64
	2010	16.88	18.68	14.88	7.24
	2015	14.96	17.03	16.87	13.20
	2020	17.64	15.74	12.18	6.11
Both					
	2000	46.31	46.50	47.19	53.98
	2005	45.45	45.67	46.30	52.05
	2010	45.63	45.83	46.57	52.24
	2015	45.04	45.31	47.69	43.12
	2020	46.82	45.28	46.17	47.47

### Age-specific PAFs

**Fig. 4:**
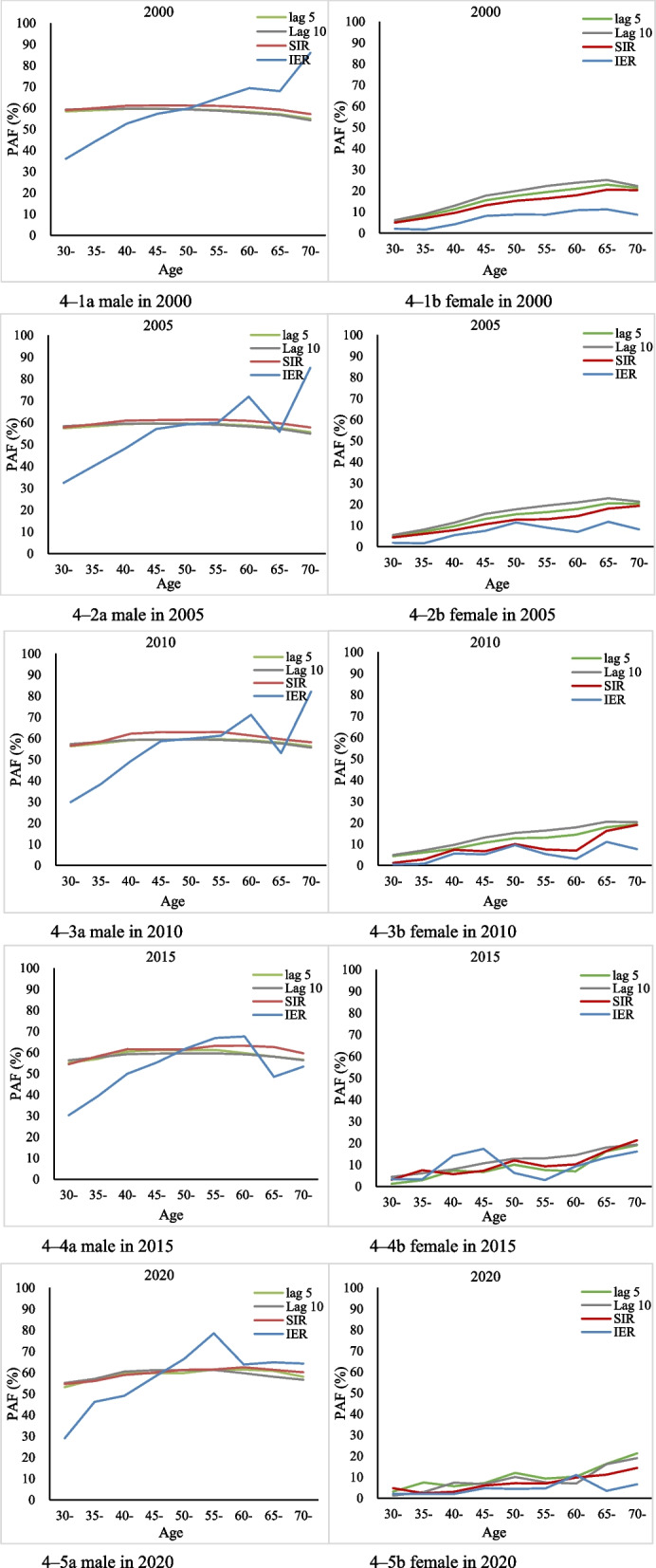
The sex- and age-specific PAFs, 2000–2020. 4–1a Male in 2000. 4–1b Female in 2000. 4–2a Male in 2005. 4–2b Female in 2005**.** 4–3a Male in 2010. Figure 4–3b Female in 2010. 4–4a Male in 2015. 4–4b Female in 2015**.** 4–5a Male in 2020. 4–5b Female in 2020

The age-specific PAFs estimated based on the lagged smoking prevalence method and the Peto-Lopez method were similar and relatively stable in men, with a slight decrease in older age groups of approximately 54–60% and 57–61%; while the dose–response relationship method showed a lower overall pattern in the younger age groups and higher in the older age groups, increasing from 29–36% in the 30–34 years to 53–86% in the 70 years and above. In particular, male PAF calculated from dose–response methods increased with age consistently in 2000, while it fluctuated slightly after 55 years and above. Female PAF increased gradually by age, from 1–5% in the 30–34 years to 14–21% in the 70 years and above; the dose–response relationship method was slightly lower than the other two methods until 2010, and it represented irregular fluctuations across age groups after 2010 (Fig. 4).

### The estimation of attributable deaths

**Fig. 5:**
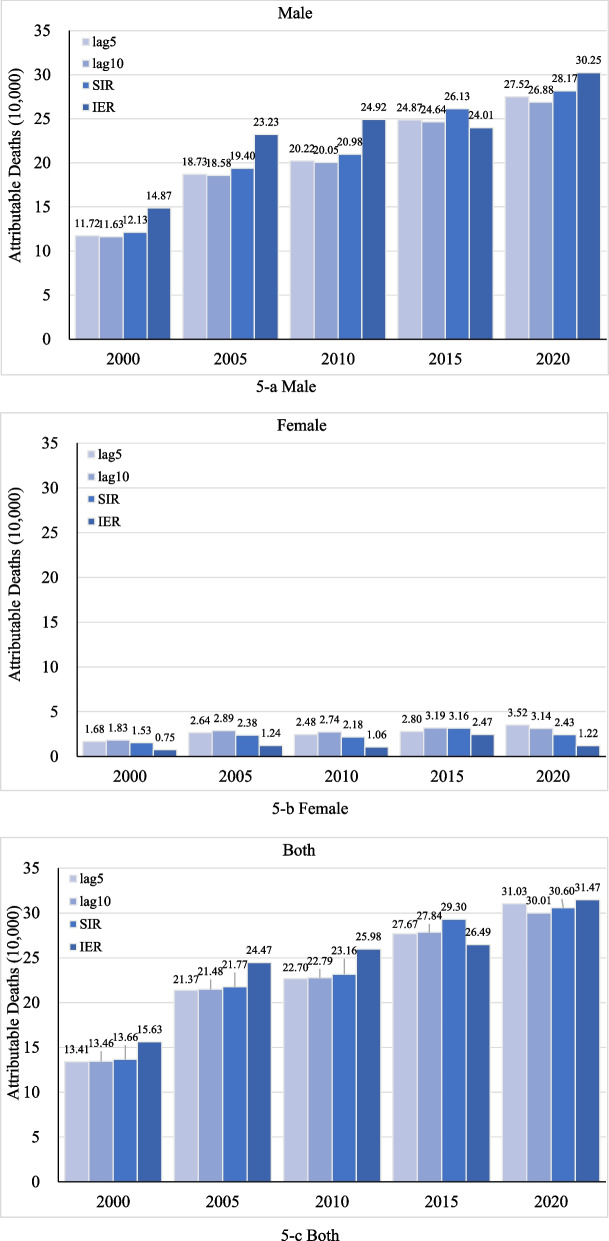
The estimation of smoking-attributable lung cancer deaths, 2000–2020. 5-a Male. 5-b Female. 5-c Both

The total number of smoking-attributable lung cancer deaths estimated by 5- and 10-year lagged smoking prevalence method, Peto-Lopez method, and dose–response relationship method were 134,100, 134,600, 136,600, and 155,300 in 2000, and then increased to 310,300, 301,100, 306,000, and 314,700 in 2020, respectively. In terms of sex, the overall changing trend was consistent in males and females, with a significant increase over time. Male deaths increased from 148,700 in 2000 to 302,500 in 2020; and female deaths increased from 7500 to 12,200, correspondingly (Fig. 5).

## Discussion

### Main results

The PAFs estimated from lagged smoking prevalence (45–47%) and Peto-Lopez method (46–47%) were comparable and both were lower than estimates from dose–response method (47–58%). Male PAFs estimated from the lagged smoking prevalence and Peto-Lopez methods rose slightly over time, whereas the estimates based on dose–response method decreased, except for showing minimum PAFs in 2015. For females, dose–response relationship-derived PAFs were lowest compared with other two methods, with maximum PAFs in 2015.

### Comparison of PAF estimations based on the three methods

#### Smoking prevalence lagged method is consistent with previous results

Since 1990s, PAFs estimated by lagged smoking prevalence and non-lagged smoking prevalence have been similar, with male PAFs ranging from 50 to 60% and female PAFs ranging from 10 to 20%. The estimates were also close to previous studies. In 1998, Liu et al. [32] estimated that Chinese male and female PAFs of smoking-caused lung cancer were 52.3% and 19.4% among population aged 35–69 years based on a national case–control study including millions of participants. In 2005, Gu et al. [33] estimated the PAF of smoking-attributable lung cancer deaths in Chinese aged 40 years and older based on 169,871 cases from the Chinese Hypertension Survey study, and the results showed that the male and female PAFs were 50.6% and 14.8%, respectively. In 2021, Li et al. [34] estimated PAFs of smoking-attributable lung cancer deaths for Chinese males aged 40–54, 55–69, and 70 years and above based on a large-scale study of smoking and cancer in an Asian population, with PAFs of 59.09%, 63.12% and 60.08%, respectively; and 3%, 7.5% and 12.71% for Chinese females, respectively.

#### The Peto-Lopez method has stable evaluations for males and lower estimates for females

Generally, PAFs for smoking-caused lung cancer deaths estimated from Peto-Lopez method in males were consistent with previous studies, while the PAFs in females (12–18%) were much lower than in previous studies (30–40%). For example, in 2004, WHO estimated the PAFs for smoking-caused lung cancer in the Chinese population aged 30 years and above to be 55.56% and 31.43% for men and women, respectively [35]. In 2013, Liu Yunning et al. [36] estimated that the PAFs for smoking-caused lung cancer deaths by using Peto-Lopez method in the Chinese population were 67.17% and 40.32% for males and females, respectively.

Liu et al. [37] used RR from GBD 2013, and the lung cancer mortality of nonsmokers was calculated from the China Kadoorie Biobank (CKB) study, with uniform RR among both sexes in all age groups, and the final male and female RR per unit of SIR were 22.51 and 14.09. Changes among PAFs by Peto-Lopez method can be attributable to the age-specific variations of RR per unit of SIR. The male and female RRs per unit of SIR were lower at younger ages while climbing to higher levels when they were older. This change is consistent with the pattern of cumulative effects of smoking with increasing age. However, when we use fixed RR, the age effect could be neglected and the attributable risk might be overestimated among younger population.

#### The dose–response method seems to drive higher estimation

Up to now, our estimates derived from dose–response method could only be compared with GBD studies due to lack of other studies. The estimated PAFs of males and females in the studied years were significantly higher in GBD 2019 study. The overall PAFs were 47.47% (male 65.26%, female 6.11%) in 2020, while the PAFs from GBD 2019 were 64.9% (male 82.3%, female 26.2%). The sex- and age-specific PAFs showed similar increasing trend, with more smoothed changes over ages in GBD. We infer that the overall discrepancies could be caused by dose–response RR as shown in our previous study [24]. The age- and sex-specific trend could be explained by the cumulative effect on older adults and the fluctuation in our study is mainly caused by smaller sample size in higher age groups. These also reflect that the dose–response methods have higher requirements of data quality.

#### Comparison of the three methods

Our study showed that among three methods used to estimate the PAFs of smoking-related lung cancer deaths in China, the dose–response relationship method yielded the highest estimates with the Peto-Lopez method being the second lowest and the lagged method for smoking prevalence being the lowest. We thought the major discrepancies came from RR estimation. The dichotomous RR of smoking-attributable lung cancer remains stable over time. Accordingly, the RRs per unit of SIR, which is back-calculated based on the dichotomous RRs and smoking prevalence were stable as well. Thus, PAFs estimated based on the lagged smoking prevalence method and the Peto-Lopez method remain relatively stable across age groups. While the dose–response relationship method quantified the cumulative risk of smoking over age and time, allowing exposure and risk to reflect on the changes in smoking behavior and effect of tobacco control strategies. In dose–response relationship method, the risk of developing lung cancer increases as the accumulation of PYs smoked by a person [38, 39] and decreases as the years of quitting smoking extended [40], and by using sex- and age-specific PYs and QYs exposures as weight, the RRs were distinguished by accumulative exposures among individuals. Overall, the PAF showed age-specific trend, with lower PAFs among younger adults and higher PAFs among older adults. According to *Quitting Smoking: Surgical Report* published by Disease Prevention and Control Center in the United States in 2020, smoking cessation reduces the risk of reproductive, cardiovascular disease, chronic obstructive pulmonary disease, and 12 types of cancer [41]. Our study also supports that the health risks of smoking decrease gradually as the duration of smoking cessation increases [42].

Among the three methods, the former two methods are easy to use, however, the accumulative effects among different ages and changes have not been fully considered. While the dose–response method uses more comprehensive perspective of smoking exposure as PYs integrate both duration and dose of smoking exposure and QYs consider the deduction risk [9]. It should be noticed that when using dose–response method, the country-specific estimation is necessary, especially for the countries with high exposure to other competing risks such as fossil fuel use and cooking. We also call for more transparent, accessible clarifications for methods and data sources in GBD studies to promote study dissemination.

### Innovativeness and implications

This study estimated the PAFs and smoking-attributed lung cancer deaths in Chinese adults based on first-hand survey data and up-to-date evidence in the Chinese population, providing concrete evidence for smoking-attributable lung cancer deaths estimation in China. Moreover, calculating and comparing smoking-attributable lung cancer deaths based on three major methods provides valuable reference for the methodology development of smoking-attributable disease burden estimation. This study indicates that the dose–response relationship method has potential to reflect the smoking-attributable implications more reasonably with reliable and stable data source.

### Limitations

First, insufficient data in smoking exposure led to fluctuations across age groups, for instance, PYs of lower or higher age groups were missing due to small sample size. To solve this, we used data from adjacent age group as a substitute. Second, we used second-hand data for dose–response RR curves fitting, without fully consideration of other confounding factors and interaction or mediation effects with air pollution or occupational factors. We used adjusted RR to fit dose–response RR curves which could mitigate most potential effects. Third, this study focused on the attributable deaths caused by active smoking only and did not include passive smoking, which may lead to underestimates. Fourth, we only estimated the lung cancer deaths caused by smoking due to the very limited dose–response relationship studies in the Chinese populations, lack of general perspectives on smoking-caused disease burden among all causes.

## Data Availability

Data are available upon reasonable request. The data sets used in this study were part of publicly accessible on the official website of Chinese Center for Disease Control and Prevention. Data can be accessed by reasonable application.
